# Pneumococcal nasopharyngeal carriage, serotype distribution, and vaccination status in hospitalized children in Latvia: A cross-sectional study during the post-PCV13, pre-PCV15/PCV20 transition period

**DOI:** 10.1371/journal.pone.0359297

**Published:** 2026-09-25

**Authors:** Hedija Cupeca, Indra Zeltina, Anita Villerusa, Larisa Savrasova, Ilze Grope, Maksims Mukans, Juris Kibilds, Angelika Krumina, Peteris Tretjakovs, Ludmila Viksna

**Affiliations:** 1 Department of Pediatrics, Riga Stradins University, Riga, Latvia; 2 Department of Pediatrics, Children’s Clinical University Hospital of Latvia, Riga, Latvia; 3 Department of Infectology, Riga Stradins University, Riga, Latvia; 4 Institute of Public Health, Riga Stradins University, Riga, Latvia; 5 Institute of Food Safety, Animal Health and Environment BIOR, Riga, Latvia; 6 Department of Human Physiology and Biochemistry, Riga Stradins University, Riga, Latvia; Bangladesh Council of Scientific and Industrial Research, BANGLADESH

## Abstract

*Streptococcus pneumoniae* remains a leading cause of invasive disease in children, and nasopharyngeal carriage surveillance is essential for monitoring vaccine-driven changes in serotype distribution. However, carriage data from the Baltic states are scarce. We conducted a cross-sectional study to assess pneumococcal carriage prevalence, serotype distribution, and associated factors in 321 hospitalized children aged 0–18 years at the Children’s Clinical University Hospital of Latvia between January 2021 and September 2022, a period representing the post-PCV13, pre-PCV15/PCV20 era. Pneumococcal carriage was detected by PCR targeting the *lytA* and *sp2020* genes; serotyping was performed on PCR-positive samples. Overall carriage prevalence was 21.8% (70/321; 95% CI: 17.6–26.6%). Of the PCR-positive samples, 78.6% (55/70) were non-typeable and only 21.4% (15/70) yielded identifiable serotypes. Among these 15 typeable isolates, the most frequently detected serotypes were 20, 13, and 48, appearing in complex combinations, followed by 15A/15F, 23B1/23B, and 09N/09L—none of which are covered by PCV13; given the small number of typeable isolates, serotype findings are exploratory. PCV vaccination coverage was 63.2% (203/321), with PCV10 (Synflorix) predominating. No significant association was found between vaccination status and carriage prevalence (vaccinated: 25.6% vs. unvaccinated: 16.7%; p = 0.138). Children aged 1–5 years had the highest carriage prevalence at 30.9%. These data constitute the first comprehensive pneumococcal carriage assessment from the Baltic region during this transitional period and may serve as a reference for evaluating the impact of PCV15 and PCV20 following their introduction.

## Introduction

*Streptococcus pneumoniae* (pneumococcus) remains a leading cause of invasive pneumococcal disease (IPD), pneumonia, and acute otitis media in children worldwide [1,2]. The introduction of pneumococcal conjugate vaccines (PCVs) has substantially reduced the burden of vaccine-type (VT) disease; however, this reduction has been accompanied by the emergence of non-vaccine-type (NVT) replacement serotypes in many settings [3,4]. Epidemiological differences among serotypes influence their pathogenic potential and inform vaccine formulation strategies [5].

Latvia introduced PCV vaccination into the national immunization program, with PCV10 (Synflorix) as the predominantly used vaccine. By 2021–2022, PCV13 had been available for several years, creating a post-PCV13 epidemiological landscape characterized by reduced circulation of VT serotypes and progressive emergence of NVT replacements [6,7]. European surveillance data indicate that serotypes 8, 19A, 22F, and 11A are among the most common causes of IPD in the post-PCV13 era [8], and systematic reviews have documented regional variations in replacement patterns [7].

Two next-generation vaccines have since been developed: PCV15 (Vaxneuvance, adding serotypes 22F and 33F to the PCV13 formulation) and PCV20 (Prevnar 20, adding serotypes 8, 10A, 11A, 12F, 15B, 22F, and 33F) [9,10]. These vaccines target important NVT serotypes that emerged in the post-PCV13 period [11,12]. The 2021–2022 period thus represents a critical epidemiological window—after PCV13 implementation but before widespread PCV15/PCV20 adoption—and baseline data from this interval are needed to evaluate the eventual impact of these newer vaccines [13,14].

Nasopharyngeal carriage studies are valuable for several reasons: first, carriage precedes and predicts invasive disease; second, the nasopharynx serves as a transmission reservoir; and third, carriage reflects vaccine-driven selective pressure more rapidly than invasive disease surveillance alone [15,16]. Studies in hospitalized children capture both colonization and potential disease-associated isolates, offering insight into transmission dynamics as well as clinical relevance [17].

Despite the importance of carriage surveillance, comprehensive data from the Baltic states during this transition period have been limited. Recent surveillance of IPD in Latvia (2012–2022) identified serotype 3 as the most prevalent cause of invasive disease, with serotypes 22F and 20 emerging as important contributors [18]. However, carriage data to complement these invasive disease findings have been lacking.

The aims of this study were: (1) to determine the prevalence of *S. pneumoniae* nasopharyngeal carriage in hospitalized children in Latvia; (2) to characterize serotype distribution, with particular attention to non-typeable isolates and NVT replacement serotypes; (3) to examine associations between carriage, vaccination status, and demographic factors; and (4) to provide a baseline dataset for comparison with future post-PCV15/PCV20 surveillance.

## Materials and methods

### Study design and setting

This cross-sectional study was conducted at the Children’s Clinical University Hospital of Latvia (BKUS), the largest pediatric tertiary care facility in Latvia and the principal national referral centre for pediatric care. Enrollment took place between January 2021 and September 2022; 314 of the 321 participants (97.8%) were enrolled between December 2021 and September 2022, so the cohort predominantly reflects that period. BKUS receives patients from the capital region (Vidzeme) as well as from Kurzeme, Zemgale, and Latgale. As a tertiary referral centre, BKUS admits children with a broad spectrum of acute and chronic conditions; the implications of this single-centre, hospital-based sampling frame for the generalizability of our findings are considered in the Limitations.

### Study population

A total of 321 children were enrolled. Inclusion criteria were: (1) age 0–18 years; (2) hospitalization at BKUS during the study period; and (3) written informed consent from parents or legal guardians. Children were excluded if informed consent could not be obtained. No exclusion criteria were applied beyond this; in particular, children sampled at more than one admission were not excluded. The 321 specimens were obtained from 317 children, and all were retained in the primary analysis; restricting the analysis to one specimen per child gave a carriage prevalence of 22.1% (70/317), essentially unchanged. Participants were recruited across all participating inpatient services. The distribution of enrolling departments was as follows: emergency/admissions unit (115/321, 35.8%), general pediatrics/therapy (61/321, 19.0%), infectious diseases (57/321, 17.8%), cardiology (36/321, 11.2%), pulmonology (33/321, 10.3%), and gastroenterology (19/321, 5.9%). Because children admitted for respiratory illness may carry pneumococcus more frequently than the general pediatric population, a sensitivity analysis restricting the cohort to non-respiratory admissions (i.e., excluding the pulmonology and infectious diseases services) was performed, as described below.

According to Eurostat, the total population of children aged 0–17 years in Latvia during 2021–2022 was approximately 340,000–350,000 [19]. For an estimated carriage prevalence of 21.8%, a sample size of 321 provides a margin of error of ±4.5% at the 95% confidence level, which is consistent with sample sizes reported in comparable European carriage studies [15,16,20].

### Sample collection

Nasopharyngeal or oropharyngeal swabs were collected from each participant into sterile swab tubes containing Amies clear solid transport medium (Meus S.r.l., Piove di Sacco, Italy). Specimens were frozen immediately after collection and stored at −20 °C until DNA extraction. Overall, 229 of 321 specimens (71.3%) were nasopharyngeal and 92 (28.7%) were oropharyngeal; the anatomical site sampled was determined by clinical indication and department protocols. Samples were obtained as part of routine clinical care for respiratory symptoms or infections. Because nasopharyngeal and oropharyngeal specimens may differ in sensitivity for pneumococcal detection, carriage prevalence was compared between the two specimen types (Results), and specimen type was included among the factors examined in relation to carriage.

### Laboratory methods

Molecular analyses were performed at the Institute of Food Safety, Animal Health and Environment “BIOR” (Riga, Latvia). All testing was performed directly on swab specimens, without a culture or enrichment step. For nucleic acid extraction, the swab head was detached and placed in 1 mL of phosphate-buffered saline, vortexed for 10 s, and centrifuged at 12,000 × g for 5 min; the resulting pellet was then processed manually using the InstaGene Matrix (Bio-Rad Laboratories, Hercules, CA, USA) following the manufacturer’s protocol for bacteria.

*S. pneumoniae* was detected by real-time PCR targeting two pneumococcal genes, *lytA* (encoding the major autolysin) and *sp2020* (SP_2020). The *lytA* assay used the CDC primers and probe described by Carvalho et al. [21] (forward 5′-ACGCAATCTAGCAGATGAAGCA-3′, reverse 5′-TCGTGCGTTTTAATTCCAGCT-3′, probe 5′-FAM-TGCCGAAAACGCTTGATACAGGGAG-BHQ1–3′); the *sp2020* assay used the primers and probe described by Tavares et al. [22] (forward 5′-TAAACAGTTTGCCTGTAGTCG-3′, reverse 5′-CCCGGATATCTCTTTCTGGA-3′, probe 5′-FAM-AACCTTTGTTCTCTCTCGTGGCAGCTCAA-BHQ-3′; amplicon 155 bp). All reactions were performed using Luminaris Color Probe qPCR Low ROX Master Mix (Thermo Fisher Scientific, Waltham, MA, USA), with primer and probe concentrations of 0.3 μM and 0.2 μM, respectively, on a ViiA 7 real-time PCR instrument (Applied Biosystems, Foster City, CA, USA). Cycling conditions were 50 °C for 2 min and 95 °C for 10 min, followed by 45 cycles of 95 °C for 15 s and 60 °C for 1 min. Positive and negative controls were included in every run, with DNA from an in-house pneumococcal isolate serving as the positive control.

A specimen was classified as PCR-positive when either target was detected, applying a per-assay cut-off of a cycle threshold (Ct) of ≤40 within 45 amplification cycles. Sixty-three of the 70 positive specimens met this cut-off; in the remaining seven a target was detected only marginally later (*lytA* Ct 40.6–43.7). These seven were retained in the primary analysis and were examined in a sensitivity analysis (Results); all seven were non-typeable, so they do not affect any serotype-specific finding. Because *lytA* may occasionally be detected in non-pneumococcal streptococci, *sp2020* was included as a second, species-specific target. In this cohort all 70 PCR-positive specimens were *lytA*-positive and 41 (58.6%) were additionally *sp2020*-positive; the interpretation of specimens positive for *lytA* alone is considered in the Results and Discussion.

Molecular serotyping was attempted directly on all PCR-positive specimens using the sequencing-based “sequetyping” approach described by Gonzales-Siles et al. [23]. A 1061-bp region of the capsular *cpsB* gene was amplified using two primer pairs (cps1 5′-GCAATGCCAGACAGTAACCTCTAT-3′ with wzh-mid1-R 5′-TGGCGTGATTCCCAACATCAA-3′; and cps2 5′-CCTGCCTGCAAGTCTTGATT-3′ with wzh-mid-F 5′-CCCTTAATGATAGTCGTTATGC-3′). Consensus sequences of the *cpsB* amplicon were compared by BLAST similarity search against the SeroBA reference database [24] (downloaded 18 August 2022). Following Gonzales-Siles et al., a sequence was assigned to a specific serotype when the BLAST similarity was ≥ 99% and the similarity to the next best matching reference sequence was at least 1% lower. Where several database entries matched within <1% of one another, or where the best-matching entry was itself annotated as a serotype complex, the result is reported as a serogroup/serotype complex (for example, the 20/13/48 complex), because these serotypes cannot be resolved from the *cpsB* fragment alone. Specimens in which a pneumococcal target was detected but no *cpsB* sequence could be assigned were classified as non-typeable (recorded as “Netipejams”). When more than one serotype was indicated in a single specimen, all were recorded, potentially representing co-colonization by multiple strains.

### Data collection

Demographic and clinical data were collected using a structured questionnaire and medical record review. Variables included: age, sex, geographic region, vaccination history (PCV type and number of doses), clinical information (hospitalization department, ARVI symptoms, pneumonia diagnosis, otitis media history), household factors (number of children, daycare attendance, household smoking), antibiotic use, and COVID-19 status.PCV vaccination status was ascertained from parental report together with available medical and immunization records, and was classified as vaccinated (≥1 documented PCV dose), unvaccinated, or unknown. PCV coverage was calculated as the proportion of all enrolled children classified as vaccinated. Recruitment took place during the study period defined above (Study design and setting).

### Statistical analysis

Categorical variables are summarized as counts and percentages and continuous variables as medians with interquartile ranges (IQR) or means with standard deviations. Carriage prevalence was calculated as the proportion of PCR-positive specimens among all enrolled participants, and 95% confidence intervals (CIs) for proportions were estimated using the Wilson method. Serotype distribution was described among PCR-positive specimens. Associations between categorical variables were examined using the chi-square test or Fisher’s exact test, as appropriate. Age was categorized into three groups: 0–1 years, 1–5 years, and >5 years. To assess whether the association between vaccination status and carriage was confounded by age and daycare attendance, a multivariable logistic regression model was fitted with carriage as the outcome and vaccination status, age group, and daycare attendance as covariates; adjusted odds ratios (aOR) with 95% CIs are reported. Additional analyses examined the association of carriage with specimen type (nasopharyngeal vs oropharyngeal), current and recent (previous-month) antibiotic use, and COVID-19 status. Cycle-threshold values were compared between typeable and non-typeable specimens using the Mann-Whitney U test. Sensitivity analyses re-estimated carriage prevalence after excluding respiratory admissions (pulmonology and infectious diseases), and under two alternative definitions of PCR positivity: restriction to specimens meeting the Ct ≤ 40 cut-off, and a stricter dual-target definition requiring detection of both targets. Statistical significance was set at two-sided p < 0.05. Analyses were performed using SPSS version 27.0 (IBM Corporation, Armonk, NY, USA); figures were generated using Python (matplotlib).

### Ethics statement

This study was performed in accordance with the Declaration of Helsinki. Ethical approval was obtained from the Riga Stradins University Research Ethics Committee (approval number 6–3/20, dated 29 November 2018). Institutional permission was granted by the Children’s Clinical University Hospital of Latvia (dated 22 January 2018). Written informed consent was obtained from parents or legal guardians of all participating children.

## Results

### Study population characteristics

A total of 321 children were enrolled (Table 1). The median age was 71 months (IQR: 25.0–140.0; range: 0–215 months). Children under 5 years comprised 44.2% (142/321) of the cohort. Sex distribution was balanced, with 166 males (51.7%) and 155 females (48.3%). The majority of participants were from the Vidzeme region (248/320, 77.5%). Enrollment occurred predominantly during winter (257/321, 80.1%) and spring (58/321, 18.1%).

**Table 1:** Study population characteristics (n = 321).

Characteristic	Value
**Total enrolled**	321
**Age, months**	
Median (IQR)	71.0 (25.0–140.0)
Mean ± SD	84.6 ± 66.4
Range	0–215
**Age groups**	
0–1 years	45 (14.0%)
1–5 years	97 (30.2%)
>5 years	179 (55.8%)
**Sex**	
Male	166 (51.7%)
Female	155 (48.3%)
**Geographic region**	
Vidzeme	248 (77.5%)
Zemgale	45 (14.1%)
Latgale	14 (4.4%)
Kurzeme	13 (4.1%)
**Season of enrollment**	
Winter	257 (80.1%)
Spring	58 (18.1%)
Autumn	6 (1.9%)
**Specimen type**	
Nasopharyngeal	229 (71.3%)
Oropharyngeal	92 (28.7%)

### Pneumococcal carriage prevalence

Overall, *S. pneumoniae* carriage, as detected by PCR, was identified in 70 of 321 children (21.8%; 95% CI: 17.6–26.6%). The remaining 251 children (78.2%) were PCR-negative. Carriage prevalence was similar for nasopharyngeal (50/229, 21.8%; 95% CI 17.0–27.6%) and oropharyngeal (20/92, 21.7%; 95% CI 14.5–31.2%) specimens (p = 0.99), indicating that combining specimen types did not materially bias the overall estimate. In a sensitivity analysis excluding respiratory admissions (pulmonology and infectious diseases services), carriage prevalence was essentially unchanged (52/231, 22.5%; 95% CI 17.6–28.3%). Among the 69 PCR-positive specimens with a quantifiable lytA cycle-threshold (Ct) value, the median Ct was 33.1 (IQR 27.9–37.4; range 20.9–44.5), and 26 (38%) had a lytA Ct above 35, indicating that many carriage specimens contained low bacterial DNA loads.

### Serotype distribution

**Fig 1:**
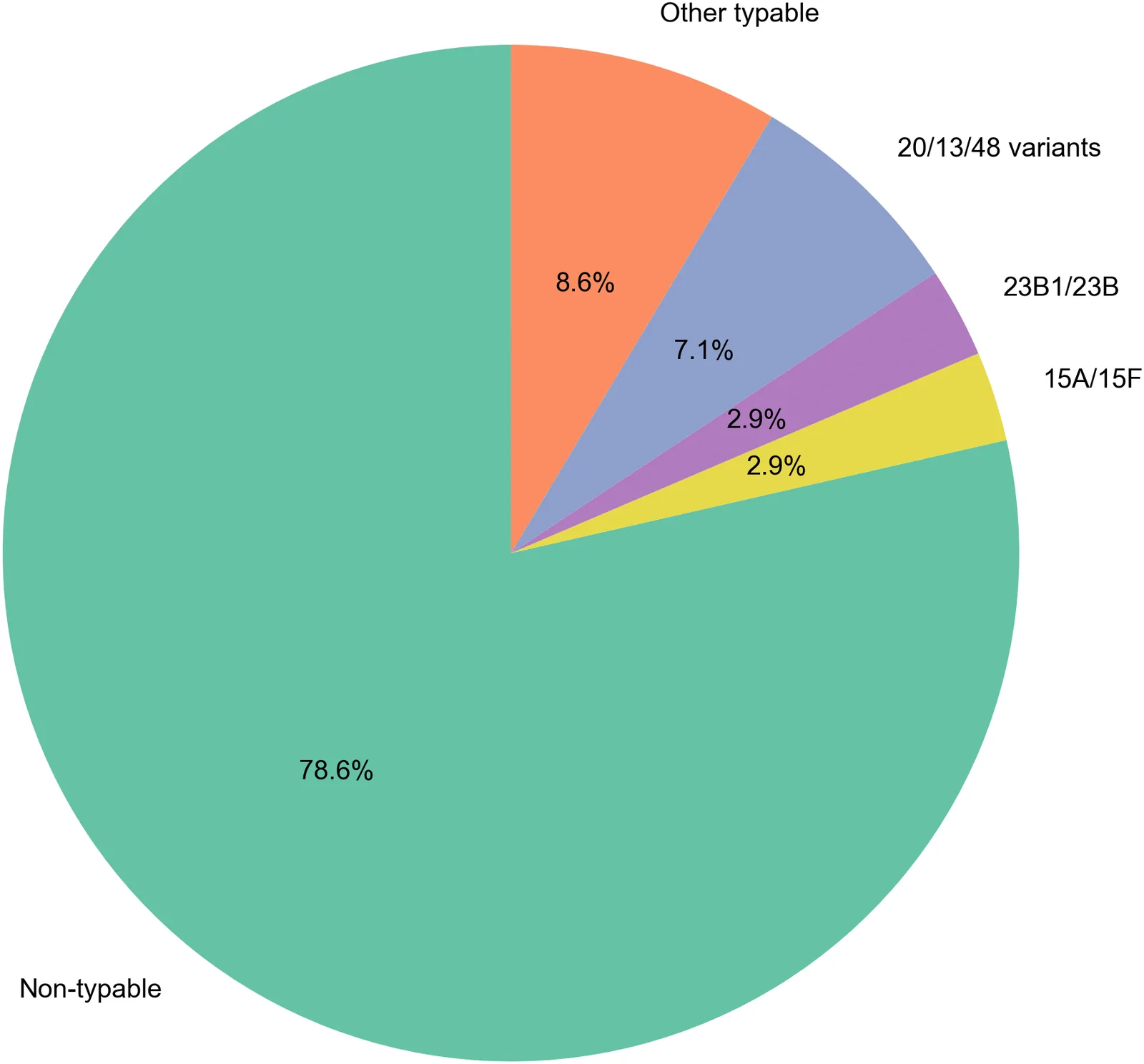
Serotype distribution among PCR-positive samples (n = 70). Non-typeable isolates accounted for 78.6% of all PCR-positive samples. Among typeable serotypes, combinations containing serotypes 20/13/48 were the most common (7.1%), followed by 15A/15F and 23B1/23B (2.9% each).

Among the 70 PCR-positive samples, 55 (78.6%) were classified as non-typeable and only 15 (21.4%) yielded identifiable serotypes (Table 2, Fig 1). Because so few specimens were typeable, the serotype distribution described below is exploratory and is not intended to represent the true distribution of circulating pneumococcal serotypes in this population; these findings are severely underpowered for serotype-specific inference and should be interpreted with caution.

**Table 2:** Pneumococcal carriage and serotype distribution among PCR-positive samples (n = 70).

Category	n	% of PCR-positive	95% CI
Non-typeable	55	78.6	67.6–86.6
Typeable serotypes	15	21.4	13.4–32.4
20/13/48 (complex combinations)	5	7.1	3.1–15.7
15A/15F	2	2.9	0.8–9.8
23B1/23B	2	2.9	0.8–9.8
24B	1	1.4	0.3–7.7
23F	1	1.4	0.3–7.7
47F/35F	1	1.4	0.3–7.7
09N/09L	1	1.4	0.3–7.7
6	1	1.4	0.3–7.7
18F/11D/11A	1	1.4	0.3–7.7

Typeability was closely related to the molecular signal. All 15 typeable specimens were positive for both *lytA* and *sp2020*, whereas none of the 29 specimens positive for *lytA* alone could be assigned a serotype (15/41, 36.6% vs 0/29, 0%; p < 0.001). Typeable specimens also showed lower *lytA* Ct values than non-typeable specimens (median 28.8, IQR 27.2–33.4 vs median 34.4, IQR 29.0–37.8; p = 0.080), indicating that serotyping success was concentrated among specimens with higher bacterial DNA load. Carriage estimates were robust to the definition of positivity: restricting positives to those meeting the Ct ≤ 40 cut-off gave a prevalence of 19.6% (63/321; 95% CI 15.7–24.3%), and a stricter dual-target definition requiring detection of both targets gave 12.8% (41/321; 95% CI 9.6–16.9%), with the proportion of non-typeable specimens falling from 78.6% to 63.4% (26/41). All seven specimens excluded by the Ct ≤ 40 cut-off were non-typeable.

Complex serotype combinations were frequently observed, with serotypes 20, 13, and 48 appearing together in various combinations in 5 samples. When individual serotypes were extracted from these complex results, the most frequently detected were serotypes 20, 13, and 48 (5 isolates each), followed by 09N and 09L (3 isolates each), 15A/15F and 23B1/23B (2 isolates each), and 24B, 23F, 47F, 35F, 6, 18F, 11D, and 11A (1 isolate each).

### Vaccination status and association with carriage

**Fig 2:**
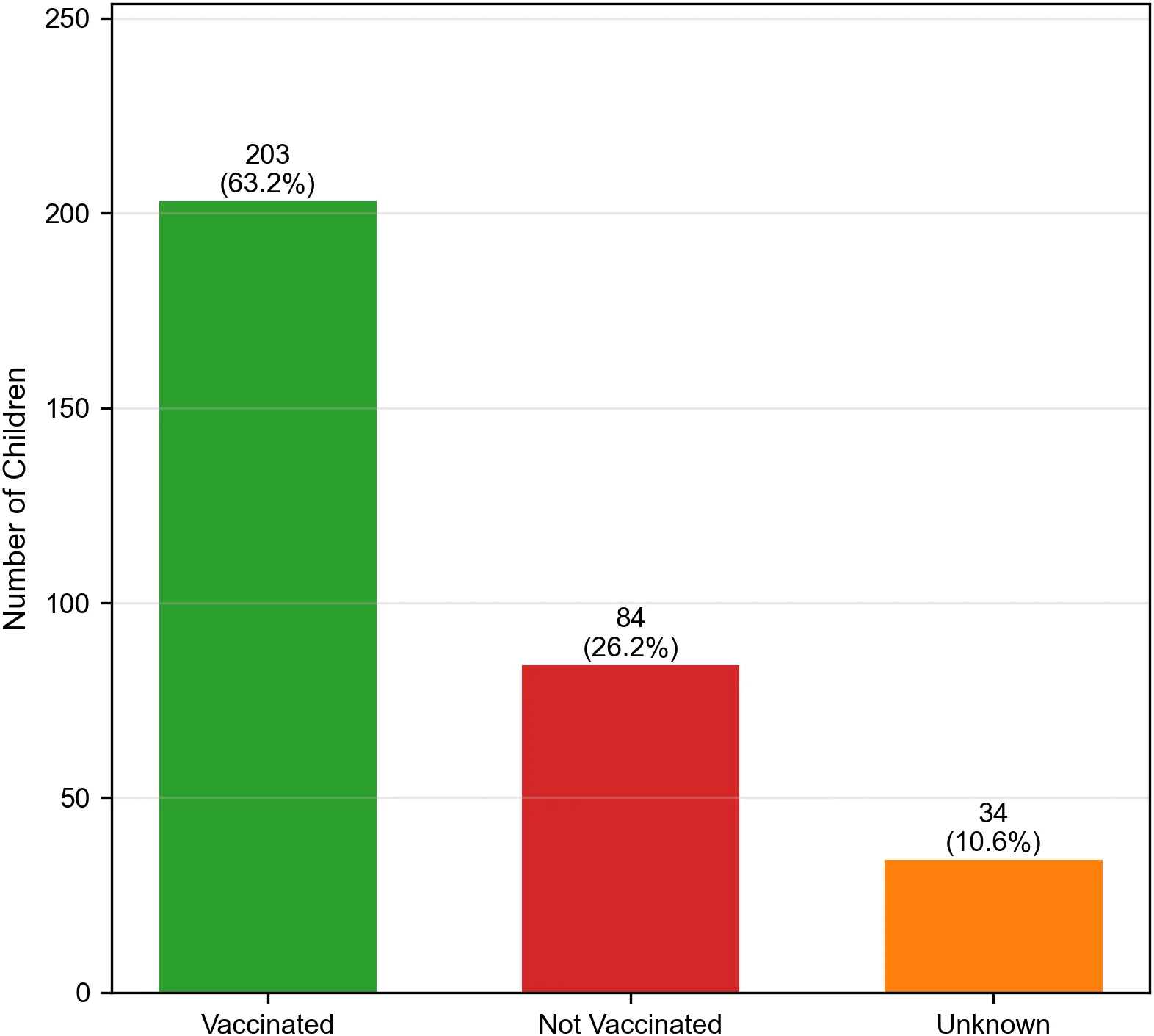
PCV vaccination coverage in the study population (n = 321). PCV10 (Synflorix) was the predominant vaccine among vaccinated children (167/203, 82.3%).

PCV vaccination coverage was 63.2% (203/321); 84 children (26.2%) were unvaccinated and vaccination status was unknown for 34 (10.6%). Among the 203 vaccinated children, the documented vaccine was PCV10 (Synflorix) in 167 (82.3%), the earlier 7-valent formulation (PCV7) in 22 (10.8%), and PCV13 in 8 (3.9%), with the valence undetermined for 6 (3.0%). These categories are mutually exclusive and reflect the most recently documented product; the small numbers of PCV7 and PCV13 recipients are consistent with changes in the national program over the study children’s lifetimes. Most vaccinated children (161/203, 79.3%) had received ≥3 doses (Table 3, Fig 2).

**Table 3:** Vaccination status and association with pneumococcal carriage.

Variable	n	%	p-value
**PCV vaccination status**			
Vaccinated	203	63.2	
Not vaccinated	84	26.2	
Unknown	34	10.6	
**PCV type (among vaccinated)**			
PCV10 (Synflorix)	167	82.3	
PCV7 (Prevenar)	22	10.8	
PCV13 (Prevenar 13)	8	3.9	
Unknown valence	6	3.0	
**Number of PCV doses**			
1 dose	14	6.9	
2 doses	28	13.8	
3 + doses	161	79.3	
**Carriage by vaccination status**			
Vaccinated (carriers/total)	52/203	25.6	
Not vaccinated (carriers/total)	14/84	16.7	0.138

Carriage prevalence was higher among vaccinated than unvaccinated children (52/203, 25.6% vs 14/84, 16.7%), but this difference was not statistically significant (p = 0.138). The association was further attenuated after adjustment: in a multivariable logistic regression that included age group and daycare attendance, vaccination was not independently associated with carriage (aOR 1.45, 95% CI 0.71–2.95; p = 0.31). Neither age group (1–5 vs 0–1 years: aOR 1.84, 95% CI 0.65–5.19; > 5 vs 0–1 years: aOR 0.97, 95% CI 0.41–2.33) nor daycare attendance (aOR 1.00, 95% CI 0.48–2.08) was independently associated with carriage, supporting the interpretation that the crude vaccinated–unvaccinated difference reflects confounding rather than a causal effect of vaccination on carriage.

### Age-specific carriage prevalence

**Fig 3:**
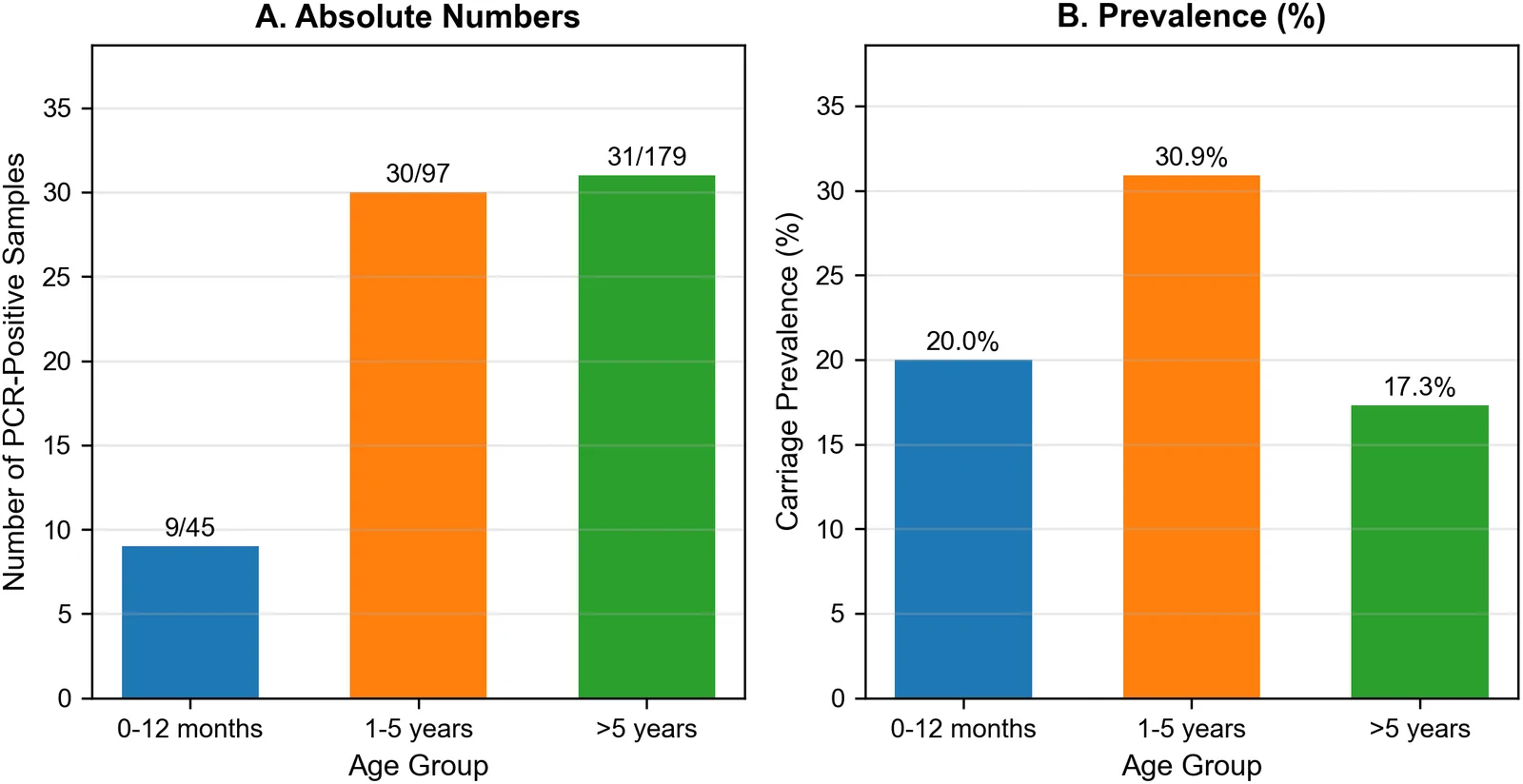
Pneumococcal carriage prevalence by age group. Panel A: absolute numbers of PCR-positive samples by age group. Panel B: carriage prevalence (%). The 1–5 year age group had the highest prevalence at 30.9%.

The highest carriage prevalence was observed in children aged 1–5 years (30.9%, 30/97), followed by children aged 0–12 months (20.0%, 9/45) and those older than 5 years (17.3%, 31/179) (Fig 3).

### Clinical and household factors

**Fig 4:**
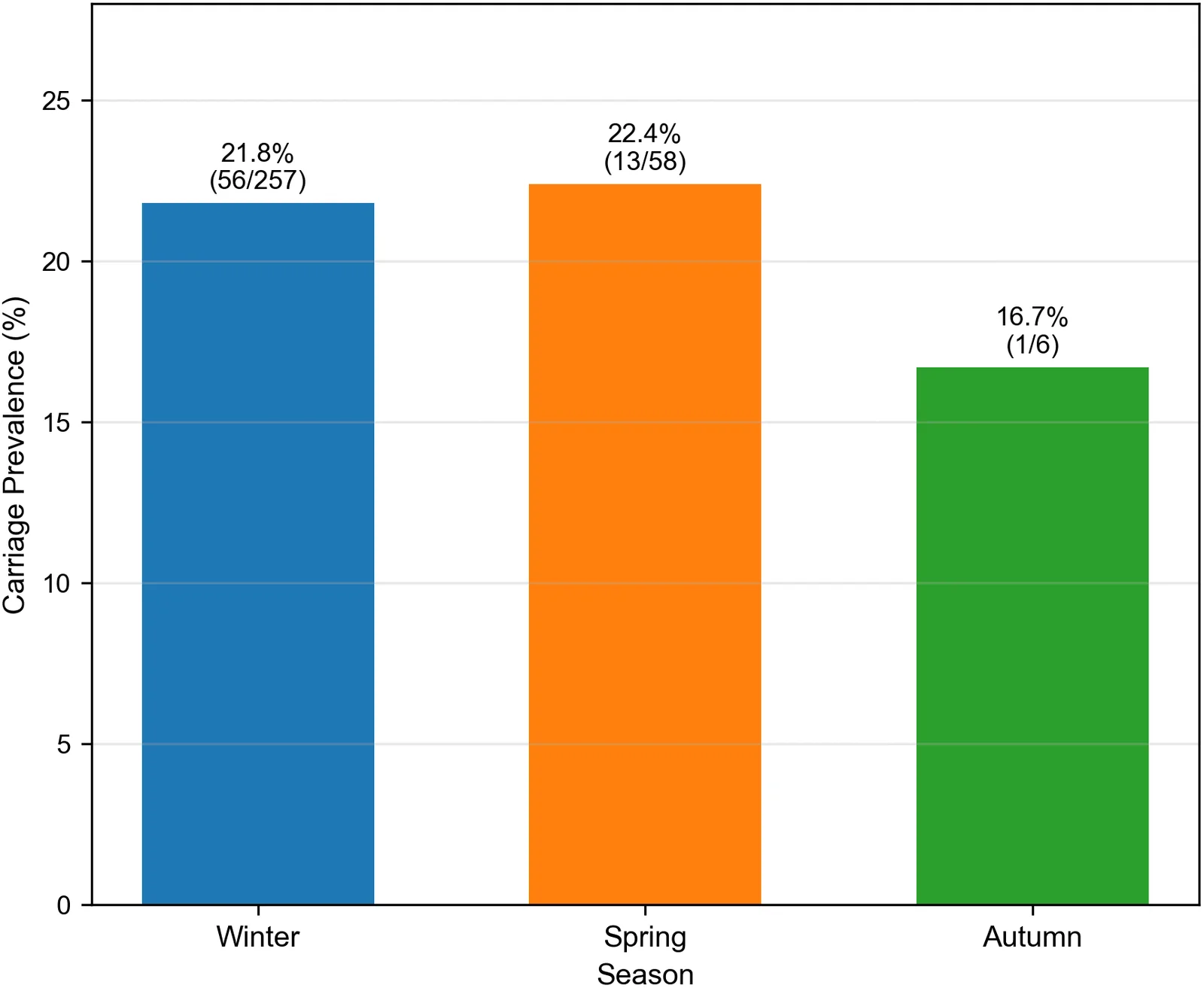
Pneumococcal carriage by season of enrollment. The majority of enrollments (80.1%) occurred during winter months, which also had the highest carriage rates.

Table 4 presents the distribution of clinical and household factors in the study cohort. ARVI symptoms in the preceding week were reported in 58.9% (189/321) of children, otitis media in the past year in 15.6% (50/321), and pneumonia diagnosis in 3.1% (10/321). Current antibiotic use was documented in 28.0% (90/321), with amoxicillin the most frequently prescribed agent (18 cases). During the study period, 14.3% (46/321) of children tested positive for COVID-19. Carriage prevalence did not differ significantly by current antibiotic use (23/90, 25.6% in children receiving antibiotics vs 47/226, 20.8% in those not; p = 0.36); antibiotic use in the previous month was associated with a lower, though non-significant, carriage prevalence (6/50, 12.0% vs 64/271, 23.6%; p = 0.068), consistent with transient suppression of detectable colonization. Carriage was not significantly associated with concurrent COVID-19 (8/46, 17.4% in COVID-19–positive vs 62/275, 22.5% in COVID-19–negative children; p = 0.43). The majority of enrollments occurred during winter (257/321, 80.1%); carriage prevalence by season of enrollment is shown in Fig 4. Univariable associations between carriage and demographic, clinical, and household factors, with 95% confidence intervals and p-values, are summarized in Table 5. Carriage differed significantly by age group (p = 0.031) and was higher among children reporting ARVI symptoms in the preceding week (52/189, 27.5% vs 18/131, 13.7%; p = 0.003); no other factor examined was significantly associated with carriage.

**Table 4:** Clinical and household factors.

Factor	n (%)
**Age group and carriage**	
0–12 months	9/45 (20.0)
1–5 years	30/97 (30.9)
>5 years	31/179 (17.3)
**Clinical factors**	
ARVI symptoms (past week)	189 (58.9)
Pneumonia diagnosis	10 (3.1)
Otitis media (past year)	50 (15.6)
**Antibiotic use**	
Previous month	50 (15.6)
Current use	90 (28.0)
**Household factors**	
Mean children in household	1.9 (range: 0–8)
Children <5 years in household	180 (56.1)
Family member in healthcare/childcare	83 (25.9)
Household smoking	143 (44.5)

**Table 5:** Factors associated with pneumococcal carriage (univariable analysis).

Factor	n	Carriage, n (%)	95% CI	p-value
**Age group**				**0.031**
0–1 years	45	9 (20.0)	10.9–33.8	
1–5 years	97	30 (30.9)	22.6–40.7	
>5 years	179	31 (17.3)	12.5–23.5	
**Sex**				**0.829**
Male	166	37 (22.3)	16.6–29.2	
Female	155	33 (21.3)	15.6–28.4	
**Specimen type**				**0.985**
Nasopharyngeal	229	50 (21.8)	17.0–27.6	
Oropharyngeal	92	20 (21.7)	14.5–31.2	
**PCV vaccination status**				**0.101**
Vaccinated	203	52 (25.6)	20.1–32.0	
Unvaccinated	84	14 (16.7)	10.2–26.1	
**Daycare/school attendance**				**0.262**
Daycare	123	32 (26.0)	19.1–34.4	
School	136	24 (17.6)	12.2–24.9	
Neither	62	14 (22.6)	14.0–34.4	
**Current antibiotic use**				**0.358**
Yes	90	23 (25.6)	17.7–35.4	
No	226	47 (20.8)	16.0–26.6	
**Antibiotic use, previous month**				**0.068**
Yes	50	6 (12.0)	5.6–23.8	
No	271	64 (23.6)	19.0–29.0	
**COVID-19 status**				**0.433**
Positive	46	8 (17.4)	9.1–30.7	
Negative	275	62 (22.5)	18.0–27.8	
**ARVI symptoms, past week**				**0.003**
Yes	189	52 (27.5)	21.6–34.3	
No	131	18 (13.7)	8.9–20.7	
**Household smoking**				**0.372**
Yes	143	28 (19.6)	13.9–26.8	
No	177	42 (23.7)	18.1–30.5	
**Admission service**				**0.625**
Respiratory (pulmonology/infectious diseases)	90	18 (20.0)	13.0–29.4	
Non-respiratory	231	52 (22.5)	17.6–28.3	
**Season of enrollment**				**0.949**
Winter	257	56 (21.8)	17.2–27.2	
Spring	58	13 (22.4)	13.6–34.7	
Autumn	6	1 (16.7)	3.0–56.4	

Denominators vary because of missing values for some variables. 95% CIs for proportions were calculated by the Wilson method; p-values are from the chi-square or Fisher exact test comparing carriage across categories of each factor. In the multivariable logistic regression model (carriage ~ vaccination status + age group + daycare attendance), vaccination was not independently associated with carriage (aOR 1.45, 95% CI 0.71–2.95; p = 0.31).

## Discussion

This study provides what is, to our knowledge, the first comprehensive assessment of *S. pneumoniae* nasopharyngeal carriage in children from the Baltic states during the post-PCV13, pre-PCV15/PCV20 transition period (2021–2022). Several findings merit discussion.

### Non-typeable isolates

The high proportion of non-typeable isolates (78.6% of PCR-positive samples) is the most striking finding of this study. This may result from several factors: (1) low bacterial load in carriage samples, which reduces the sensitivity of conventional serotyping; (2) degradation of capsular polysaccharides during sample handling; (3) inherent limitations of the serotyping method employed; or (4) vaccine-driven selection pressure favoring non-encapsulated strains or novel capsular variants not recognized by current typing reagents [25–28]. High non-typeable rates have been increasingly reported in carriage studies from the post-PCV era, and our findings reinforce the need for improved molecular serotyping approaches—including multiplex PCR-based methods and whole-genome sequencing—which offer greater sensitivity for carriage samples with low bacterial loads [29–31]. An important alternative explanation, raised during peer review, is that some PCR-positive but non-typeable specimens—particularly those positive for *lytA* alone and with high Ct values (low bacterial load)—may not have represented true *S. pneumoniae*. Because *lytA* can occasionally be detected in non-pneumococcal streptococci, and 29 of 70 positive specimens (41%) were positive for *lytA* but not *sp2020*, we cannot exclude that a proportion of non-typeable results reflect very-low-density colonization at or below the limit of reliable capsular detection, or occasional non-specific detection, rather than typeable pneumococci that escaped serotyping. Our data are consistent with both explanations: no specimen positive for lytA alone yielded a serotype, and typeable specimens had substantially lower lytA Ct values than non-typeable ones (median 28.8 vs 34.4). Because capsular sequetyping amplifies a single 1061-bp cpsB fragment, it requires adequate template, and low-density specimens are therefore expected to fail typing even when genuinely pneumococcal. Applying a stricter dual-target definition reduced the estimated carriage prevalence to 12.8% and the non-typeable fraction to 63.4%, which brackets the plausible range for these estimates. This uncertainty reinforces the need for confirmatory approaches—dual-target positivity thresholds, culture-based confirmation where feasible, and whole-genome sequencing—in future surveillance.

### Replacement serotypes and next-generation vaccine coverage

Among the small number of typeable serotypes, all belonged to non-vaccine types (NVT). Serotypes 20, 13, and 48 were detected most frequently, often appearing in complex combinations that may represent co-colonization with multiple strains. This observation is consistent with recent IPD surveillance in Latvia, which similarly identified serotype 20 as an emerging contributor to disease burden [18]. Of note, serotype 20 is not included in either PCV15 or PCV20. Serotypes 15A/15F were detected in 2 samples; while serotype 15B is included in PCV20, the extent of cross-protection with 15A and 15F remains uncertain [32]. Serotypes 23B1/23B (2 samples) may represent serogroup 23 replacement following PCV7–PCV13-driven elimination of 23F [33]. Importantly, none of the typeable serotypes detected in our study corresponded to the primary additional targets of PCV15/PCV20 (serotypes 8, 10A, 11A, 12F, 15B, 22F, and 33F), although interpretation is constrained by the high non-typeable rate and small number of typeable isolates [11,12,38]. We emphasize that, with only 15 typeable specimens, these serotype observations are hypothesis-generating and cannot establish serotype replacement or vaccine-driven capsular selection; such inferences would require culture-based serotyping or whole-genome sequencing in a substantially larger, adequately powered sample.

### Vaccination and carriage

The observation that vaccinated children had a numerically (but not statistically significantly) higher carriage prevalence (25.6%) than unvaccinated children (16.7%; p = 0.138) is consistent with findings from prior carriage studies and likely reflects confounding by age and healthcare-seeking behavior rather than a true vaccine effect [34,35]. Latvia’s PCV coverage in the study population (63.2%) was almost exclusively PCV10, and the absence of a significant association between vaccination and overall carriage prevalence is consistent with the well-documented phenomenon of NVT serotype replacement maintaining total carriage rates despite VT reduction [36,37].

### Regional context

Limited pneumococcal carriage data exist for the Baltic states. European studies in comparable pediatric populations have reported carriage prevalences ranging from 15% to 40%, depending on age distribution, season, and vaccination coverage [20]. Our observed prevalence of 21.8% falls within this range. The European Centre for Disease Prevention and Control reported that serotypes 8, 19A, 22F, and 11A were among the most common causes of IPD in the EU/EEA in 2022 [8]. Latvia has since transitioned to PCV15 (beginning in early 2024), with PCV20 also becoming available [18]. Our pre-transition data thus provide an essential reference point for evaluating the serotype-specific effects of these newer vaccines in the Baltic region.

### Comparison with the pre-PCV13 period

A direct comparison of our findings with pre-PCV13 carriage data from Latvia is not possible, because no pneumococcal nasopharyngeal carriage survey from the pre-PCV13 period has been published for this population; that absence is precisely the surveillance gap the present study was designed to fill, and it is the reason we frame these data as a baseline for future rather than retrospective comparison. The only national dataset spanning the introduction of higher-valency conjugate vaccines is invasive pneumococcal disease surveillance covering 2012–2022 [18], which documented a decline in PCV13-type invasive disease alongside the emergence of serotypes 22F and 20 — the latter also the most frequently detected serotype among our typeable carriage isolates. Our carriage findings are therefore consistent in direction with the national invasive disease record, although carriage and invasive disease are distinct endpoints and the small number of typeable isolates precludes any quantitative inference about serotype replacement. Where pre- and post-PCV comparison has been possible elsewhere in Europe, the consistent pattern has been stable overall carriage prevalence with progressive replacement of vaccine types by non-vaccine types [7,38]; our observation of 21.8% carriage composed entirely of non-vaccine types among typeable isolates is compatible with that pattern, but cannot confirm it in the absence of a local pre-vaccine baseline. Establishing repeat carriage surveys now that PCV15 has been introduced is the only way to convert these data into a true before-and-after comparison.

### Limitations

Several limitations of this study should be acknowledged. First, and most importantly, the high non-typeable rate (78.6%) substantially limits our ability to draw serotype-specific conclusions. Second, the study population consisted of children hospitalized at a single tertiary referral centre, who may differ from the general pediatric population in comorbidity burden, antibiotic exposure, and healthcare contact; our estimate therefore applies to hospitalized children and should not be generalized to healthy children in the community. Children admitted for respiratory illness might in particular carry pneumococcus more frequently, although a sensitivity analysis excluding respiratory admissions yielded an essentially unchanged prevalence (22.5% vs 21.8%). Third, the 9-month enrollment period was skewed toward winter and spring, which may overestimate year-round carriage prevalence. Fourth, while the total sample (n = 321) provides adequate precision for estimating overall carriage prevalence, the small number of typeable isolates (n = 15) limits the statistical power of serotype-specific analyses. Fifth, the cross-sectional design precludes causal inferences regarding vaccination and carriage. Sixth, vaccination history was ascertained from parental report and medical records, introducing potential for misclassification. Finally, the COVID-19 pandemic coincided with the study period and may have influenced both healthcare-seeking behavior and pneumococcal transmission dynamics through non-pharmaceutical interventions [39,40]. In addition, both nasopharyngeal and oropharyngeal specimens were collected according to clinical indication; although these specimen types can differ in sensitivity for pneumococcal detection, carriage prevalence did not differ between them in our data, so the resulting measurement bias is likely limited. We found no significant association between carriage and either current antibiotic use or concurrent COVID-19, although recent (previous-month) antibiotic use showed a non-significant trend toward lower carriage; limited power for these subgroup analyses and residual confounding cannot be excluded. Finally, ascertainment of vaccine type from mixed record sources was incomplete for a subset of children, and the specificity of the PCR algorithm for low-load, lytA-only specimens could not be independently confirmed; both considerations warrant caution in interpreting the carriage and serotype estimates.

Despite these limitations, this study addresses an important gap in pneumococcal carriage surveillance from the Baltic region. As PCV15 and PCV20 are introduced in Latvia and neighboring countries, longitudinal follow-up studies will be necessary to evaluate their impact on both carriage and invasive disease. The high non-typeable rate underscores the need for investment in enhanced serotyping capacity, and collaboration across Baltic and European surveillance networks will be essential for monitoring the evolving pneumococcal epidemiology in this region.
